# Pharmacovigilance strategy: opportunities for cross-national learning

**DOI:** 10.1186/s13584-019-0319-3

**Published:** 2019-06-19

**Authors:** Irene Fermont

**Affiliations:** ISOP ISRAEL: The Israeli Chapter of the International Society of Pharmacovigilance and IFC Ltd, Beit Haarava32, Jerusalem, Israel

**Keywords:** Pharmacovigilance, Drug safety, Patient safety, Risk management, Multidisciplinarity, Big data, Artificial intelligence, Transparency, Real world evidence

## Abstract

The Israeli Ministry of Health has set up the foundations of a National Pharmacovigilance System. The next step is to adopt the best of the international ideas, trends and approaches which are shaping the future of pharmacovigilance. Specifically: 1) The risk management approach requires proactively preventing or minimizing risks, starting in early clinical development and extending all along the lifecycle of a pharmaceutical. 2) Drug safety is a multidisciplinary discipline where all stakeholders should be involved. 3) Clinical trials provide an ideal safety profile limited to the restrictive conditions of the trial. Only real-world data, from the post marketing period, will reveal the real risk/benefit balance for the use of a pharmaceutical in regular clinical care. 4) Artificial intelligence is needed to analyze the large amount of data collected through the post-marketing studies, electronic medical records and the internet. Many AI tools have been developed to support better use of pharmaceuticals. 5) Quality-oriented, thorough inspections and audits are critical for achieving patient safety. 6) Patients should be recognized as active players in their treatment who can, and should, have access to safety information through the major agencies’ websites.

Israel can benefit from several of its key assets to reach a higher level of pharmacovigilance: 1) Israel’s four HMOs are organized in a way that allows them to have quick and efficient dialogue with healthcare professionals and with patients. Moreover, a new project named, Big Data in Health, will pool the epidemiologic databases of the HMOs, providing precious information for understanding risk factors, detecting alerts, and developing personalized medicine. 2) Formal risk management activities have long been part of the culture of hospitals and should be applied increasingly to ensuring drug safety.

Israel has the organizational, scientific, technological and cultural resources needed to quickly overcome the challenges and go beyond its current state to build a unique pharmacovigilance system which could serve as an example for other countries.

## A considerable step: laying the foundations of a national pharmacovigilance system

Pharmacovigilance (PV) is defined as the science and activities relating to the detection, assessment, understanding and prevention of adverse effects or any other drug-related problem. The WHO^1^ established its Program [1] for International Drug Monitoring in response to the thalidomide disaster detected in 1961. Since its inception, the organization of pharmacovigilance in various countries, and regulations pertaining to it, have evolved mainly from lessons learned as a reaction to safety issues [2] that became public health issues.

This occurred in Israel, following the Eltroxin® case: a change in the excipients^2^ of Eltroxin® (levothyroxin) has led to imbalance the thyroid hormone level and therefore to a huge number of adverse reactions, mainly related to hypo or hyperthyroidism. The involvement of the media, law suits, an overwhelmed Ministry of Health, and an inadequately prepared industry, exposed a lack of sufficient regulation and structures to support pharmacovigilance. This commentary builds on the recent IJHPR article by Schwartzberg et al. [3]. which describes this event and how it led the Ministry of Health to develop a pharmacovigilance department and build a national pharmacovigilance system.

The system required the creation of a new department and a new regulation on pharmacovigilance. Lessons were quickly learned from the EU and USA, and the main source of Israeli Pharmacovigilance Procedure #6 is the European regulation [4, 5]. True pharmacovigilance would require a new nationwide culture centered on the patient.

As is the case everywhere, the main requirements of the Israeli system are aimed at the pharmaceutical industry, and the industry has understood the need to reach international standards. Continuing education and courses were implemented in several universities to train professionals and build this new discipline. Furthermore, the Israeli regulatory requirement for the role of Qualified Persons for Pharmacovigilance (QPPV) applied not only to industry, but also to each of the medical centers and HMOs.

With the latest Israeli regulation requiring the pharmaceutical industry to implement RMPs (Risk Management Plans) for high-risk products, the Israeli Ministry of Health is taking a new and important step toward international standards.

The creation of the Israeli Chapter of the ISOP (International Society of Pharmacovigilance) [6] has coincided with the setup of the new pharmacovigilance era in Israel, with the mission to foster drug safety through education and research, in full support of the Israeli regulators’ actions [7].

The basis for a sound pharmacovigilance system has been established. The next step for Israel is to reach, and possibly exceed, the level of the advanced countries. Not only does Israel have the means to build a unique model, it has various strengths in its healthcare system that can help make this happen.

## The current trends shaping the future of pharmacovigilance

The last 20 years have seen a tremendous evolution of pharmacovigilance concepts. However, adverse drug reactions remain the 4th to 5th leading causes of death in USA [8] and Europe, and their numbers are increasing. The following subjects, discussed regularly in scientific conferences and journals, are laying out the pharmacovigilance of tomorrow; they should now be adopted by Israel.

### Risk management: a change of paradigm

The risk management approach has brought a change of paradigm from passive data collection to proactive detection by the industry of potential risks and thorough evaluation of identified risks [9–11]. The approach also requires proposing actions to prevent or minimize the risks. Risk management is meant to be the common thread throughout the pharmacovigilance processes. One must always have in mind the question of how to prevent or to minimize the risk to patients. “THINK ACTION” is the motto when analyzing each individual case of adverse event, during the analysis of aggregate data in periodic reports, or when a new safety signal^3^ arises.

Risk management is holistic and should be implemented all along the life cycle of a product, from early clinical development to the post-marketing period [12] . It has taken more than a decade for this risk management approach to become the standard way of thinking for professionals in industry and authorities, in the EU, USA, and elsewhere in the world. Now, an extensive educational program must be established in Israel to achieve this paradigm shift. The several decades’ experience in risk management in Israeli medical centers, created to address any kind of medical risk, should provide fertile ground for quickly learning how to apply it in drug safety. This will necessitate that the link between risk management units in hospitals and the risk management approach to drug safety will be clearly identified and cross-fertilized.

### Multidisciplinarity

The regulations we have mentioned are designed for organising the relationships and requirements between regulatory authorities and industry. Even if risk management activities are meant to involve all stakeholders, including healthcare professionals, patients, and health funds, the obligations have weighed only on the pharmaceutical industry. Much of the necessary experience and knowledge are located within the industry, while healthcare professionals had neither obligations nor awareness of risk management plans. In reality, the direct involvement of healthcare professionals, patients, and HMOs in risk management plans has been minimal.

Pharmacovigilance has remained limited far too much to experts and to clinical pharmacologists or clinical pharmacists. It is a common understanding in other high-risk industries, such as aeronautics and nuclear power, that management of risks can only be efficient when applied by a multidisciplinary team and considered across the entire system. Healthcare systems are still lagging behind, and the healthcare professions continue to act in silos. The future of pharmacovigilance lies in its becoming truly multidisciplinary. [13, 14]

ISMP, the Institute for Safe Medication Practices [15] has been working for 30 years with the U.S. Food and Drug Administration to prevent medication errors. They have designed tools and alert systems targeted at industry, healthcare professionals, hospital staff, lay communities and government authorities. The Safe Practices Self-Assessment tools allow hospitals to identify and correct weaknesses. They illustrate how pharmacovigilance can spread beyond expert pharmacologists.

### Stamp

The System Theoretic Accident Model and Procedures (STAMP) [16], developed at MIT by Nancy Leveson, proposes a new model for analyzing a wide and complex system at the macro level, which was first applied to the aeronautics and the petroleum industries..’ *System safety is the part of system engineering that uses modeling and analysis to identify hazards and to design the system to eliminate or control them. STAMP is used as a safety engineering static and dynamic modeling and analysis approach to healthcare systems to provide a rigorous way to evaluate the efficacy of potential policy changes as a whole. System engineering techniques can be used in re-engineering the system as a whole to achieve the system goals, including both enhancing the safety of current drugs while, at the same time, encouraging the development of new drugs.’* [17]

The Vioxx issue has been analyzed in articles and theses “*to outline the interactions between the different pharmaceutical system components, identify the safety control structure in place and understand how this control structure failed to prevent the marketing of an unsafe drug which killed an estimated 27,000 people in the United States*” [18]. This new approach could impact authorities in their policy making.

### Real world evidence

It is clearly understood today that clinical trials take place under ideal conditions, and as a result clinical trials do not expose the true benefit/ risk profile of a drug. It is only during the wide and uncontrolled use of medications in the post-marketing period, that a more comprehensive benefit-risk balance is unveiled. It is a long and continuing process. This understanding has led to the need for real-world data, gathered through post-marketing studies and the use of pharmaco-epidemiologic methods. Collection of real-world data is now expanding beyond the scientific field, through the search for unstructured safety information exchanged by patients on the internet via various forums and social media.

Additionally, Electronic Medical Records (EMRs), produce a huge amount of precious data which brings a more accurate and personalized understanding of a medicine’s safety profile.

Risk minimization activities and public health decisions will be driven not only by science-based evidence but also by real-world evidence on medication utilization within specific populations, on the public health and environmental impact, and eventually on economic data.

### AI integration and big data

Gathering and integrating these real-world data, outside of the usual scientific borders, require time for analysis beyond human capacity. This is where Artificial Intelligence (AI), is now developing rapidly [19]. In pharmacovigilance, AI is involved in providing support to physicians writing prescriptions, checking the patient’s record and proposing the most appropriate treatment. AI can also monitor the patient’s adherence. AI can detect adverse events and identify patterns in screening all hospital records together with social media, literature and more.

Israel has one of the highest number of start-ups dedicated to healthcare and drug safety in the world. Some of their tools are already implemented in leading medical centers in Israel, such as The Chaim Sheba Medical Center in Tel Hashomer. Leading the innovations beyond Israel’s borders are companies such as Data2Life [20], Medaware [21], Mediseen [22], Vaica [23] – all of which are proposing solutions currently used in large hospitals in Israel or abroad. .

### Quality control

With the development of the international and harmonized regulation in pharmacovigilance, departments of inspection have been established in many countries [24]. An inspection program allows government to systematically check compliance of the pharmaceutical companies as well as medical centers running clinical trials. Industry has the obligation to perform periodic and systematic audits. This inspection programme is associated with robust enforcement penalties which dissuade industry from overlooking its obligations. In the EU, penalties for non-compliance could reach 5% of the total income of a pharmaceutical company. The Competent Authorities, are also submitted to the obligation of being audited by external and independent parties, to assess their efficiency in protecting the patients’ safety.

Around the world, the accreditation process for medical centers includes compliance with pharmacovigilance regulations. This is also true in Israel. On the other hand, pharmacovigilance inspections of pharmaceutical companies still have to be developed in Israel. An inspection program is a necessary step for enforcing compliance and building a real safety culture, while raising the quality and efficiency of the whole pharmacovigilance system. The same should apply to the Israeli Ministry of Health’s pharmacovigilance department, which should be audited by an independent party, mirroring FDA and EMA and other advanced authorities.

### Transparency

Transparency has been a cornerstone since the European regulation of 2010, and it represents a cultural revolution. From being strategic confidential data in the past, safety data are now largely shared and published in all languages through the 28 Member States in Europe. The same applies to the U.S. Food and Drug Administration. Major deficiencies following industry inspections, infringement procedures, penalties, judicial procedures, and fraud settlements with the U.S. Department of Justice, are immediately published online on the authorities’ website. This, though, refers mainly to industry.

This level of transparency has not yet reached Israel, especially in the healthcare system. The proliferation of la suits has even led to a culture of opacity. The Knesset, Israel’s parliament, is currently considering a new law to ensure strict independence between any investigative body and the healthcare system. Patients do not have easy access to their medical records, and the medical records are not always complete nor accurate. Awareness and concern regarding this issue are expressed by patients and medical associations [25–27], but unanimity is still far away. Solving this issue is crucial and will help to correct gaps and improve the whole system.

## Israel’s unique strengths and the next challenges

Israel, the Startup Nation, has the resources to overcome the challenges, with its built-in culture of entrepreneurship [28]. Israel benefits from a combination of strengths which could lead to the building of a unique system of drug safety. From a country starting late to organize its pharmacovigilance system, Israel could become a leader and an example for other developed countries, including the most advanced ones.

Overcoming these challenges relies on two main pillars: its HMOs, and the risk management units in medical centers.

### The national HMOs

For more than 20 years, the Israeli HMOs have had extensive databases including accurate epidemiological data on the whole Israeli population. This allows, potentially, the detection and analysis of safety signals as well as identification of risk factors. The HMOs’ organizational network allows bilateral exchange of information with healthcare professionals and patients. They could quickly advertise and implement public health decisions, nation-wide. However, the data owned by the four national HMOs are considered as their private property despite the impact they could have on public health. For historic reasons, the Ministry of Health has limited authority over accessing the data of the HMOs and imposing harmonized actions for risk prevention or minimization.

This asset, which is one of the most valuable of resources in Israel, is today far from being fully and effectively centralized; but the announcement in Davos by Israel’s Prime Minister heralds a groundbreaking change. In March 2018, he announced the launching of a project costing one billion shekels “to make data about the state of health of its population available to researchers and private companies” [29]. This means the merging of all the data of the HMOs.

What is presented today as a major tool for research and development and epidemiology can also prove immensely efficient for improving medication safety.

### The risk management units

In medical centers are also involved in the detection of safety concerns from healthcare products, be they pharmaceuticals, medical devices or medical practices. Even if the first objective of these units has been to manage the medical center’s legal liability for poor or fatal outcomes, they are also used in several medical centers for risk analysis and implementation of actions to prevent or minimize errors and risky situations. In addition, the remarkable Medical Simulation Center [30] of Sheba-Tel Hashomer medical center created by Prof Amitai Ziv provides a means for healthcare professionals to learn safer practices.

The long experience and culture gained by Israel in risk management, not only in healthcare, is a strong asset that can be applied to pharmacovigilance: THINK ACTION is a motto which is a daily routine in Israel.

## Conclusion

Converging voices are heard in Israel to move things forward and reach or exceed international standards in drug safety. These include the new patient safety associations [27], the new Israeli Chapter of the International Society of Pharmacovigilance [6, 7], plus voices from the pharmaceutical industry, managers of the medical centers, risk managers, general practitioners [26] and patient associations. The Ministry of Health should now make the decision to strengthen the authority and the resources of its pharmacovigilance system to ensure better patient safety.

Israel has all the organizational, scientific, technological and cultural resources to quickly overcome the challenges and build a uniquely strong pharmacovigilance system which could serve as an example for other countries.

## Data Availability

Not applicable.

## Abbreviations

HMO: Health Maintenance organization
ISOP: International Society of Pharmacovigilance
MIT: Massachusetts Institute of Technology
QPPV: Qualified Person for Pharmacovigilance
STAMP: System Theoretic Accident Model and Procedure
WHO: World Health Organization

## Footnotes

1 WHO is the directing and coordinating technical agency for health within the United Nations system. It is responsible for providing leadership on global health matters, shaping the health research agenda, setting norms and standards, articulating evidence-based policy options, providing technical support to countries and monitoring and assessing health trends

2 non active ingredients in the drug composition

3 Safety signal: information arising from one or multiple sources, suggesting a new potentially causal association, that is judged to be of sufficient likelihood to justify verificatory action
